# NDRG1 upregulation by ubiquitin proteasome system dysfunction aggravates neurodegeneration

**DOI:** 10.1186/s13041-024-01150-1

**Published:** 2024-10-23

**Authors:** Tomonori Hoshino, Atsushi Mukai, Hirofumi Yamashita, Hidemi Misawa, Makoto Urushitani, Yoshitaka Tashiro, Shu-ichi Matsuzawa, Ryosuke Takahashi

**Affiliations:** 1 Department of Neurology, Graduate School of Medicine, Kyoto University, Kyoto, 606-8503 Japan; 2 Department of Therapeutics for Multiple System Atrophy, Graduate School of Medicine, Kyoto University, Kyoto, 606-8507 Japan; 3 Department of Neurology, Japanese Red Cross Wakayama Medical Center, Wakayama, 640-8558 Japan; 4 Division of Pharmacology, Faculty of Pharmacy, Keio University, Tokyo, 105-8512 Japan; 5 Department of Neurology, Shiga University of Medical Science, Shiga, 520-2192 Japan; 6 Present Address: Neuroprotection Research Laboratories, Departments of Radiology and Neurology, Massachusetts General Hospital, Harvard Medical School, Charlestown, MA 02129 USA; 7 Yoshida-Konoe-cho, Sakyo-ku, Kyoto, 606-8501 Japan; 8 54 Shogoin-Kawahara-cho, Sakyo-ku, Kyoto, 606-8507 Japan

**Keywords:** Amyotrophic lateral sclerosis, Cell death, NDRG1, Neurodegeneration, Proteasome, Psmc4 (Rpt3)

## Abstract

Protein turnover is crucial for cell survival, and the impairment of proteostasis leads to cell death. Aging is associated with a decline in proteostasis, as the progressive accumulation of damaged proteins is a hallmark of age-related disorders such as neurodegenerative diseases, including amyotrophic lateral sclerosis (ALS). We previously discovered that the declining function of the ubiquitin-proteasome system (UPS) in motor neurons contributes to sporadic ALS pathologies, such as progressive motor neuron loss, protein accumulation, and glial activation. However, the mechanisms of UPS dysfunction-induced cell damage, such as cell death and aggregation, are not fully understood. This study used transcriptome analysis of motor neurons with UPS dysfunction and found that the expression of N-myc downstream regulated 1 (NDRG1) gets upregulated by UPS dysfunction. Additionally, the upregulation of NDRG1 induces cell death in the Neuro2a mouse neuroblastoma cell line. These results suggest that NDRG1 is a potential marker for UPS dysfunction and may play a role in neurodegeneration, such as that seen in ALS.

## Main

The cellular process of proteostasis is responsible for the constant production of proteins, degradation of the damaged ones, and maintenance of the protein quality [1]. Its disruption leads to cellular damage including cell death. Proteins are normally scavenged for degradation by the ubiquitin-proteasome system (UPS) and autophagy-lysosome pathway. During the aging process, the function of UPS in the spinal cord is impaired [2], and UPS dysfunction leads to aggregation, which is a hallmark of neurodegenerative diseases such as amyotrophic lateral sclerosis (ALS), characterized by the loss of upper and lower motor neurons in the spinal cord [3]. Decreased proteasome activity has been observed in sporadic ALS patients and a familial ALS mouse model [4, 5]. Furthermore, we previously established a strain of motor neuron-specific ubiquitin proteasome dysfunctional mice via the motor neuron-specific disruption of Psmc4 (also known as Rpt3), a subunit of the 26 S proteasome. These mice showed ALS-like pathologies, such as progressive motor neuron loss, locomotor dysfunction, and aggregation of proteins such as TDP-43 [6]. However, given the range of UPS functions, the exact mechanism of motor neuron damage owing to UPS dysfunction remains undeciphered. In this study, we aimed to identify a novel regulator of motor neuron damage induced by UPS dysfunction.

**Fig. 1:**
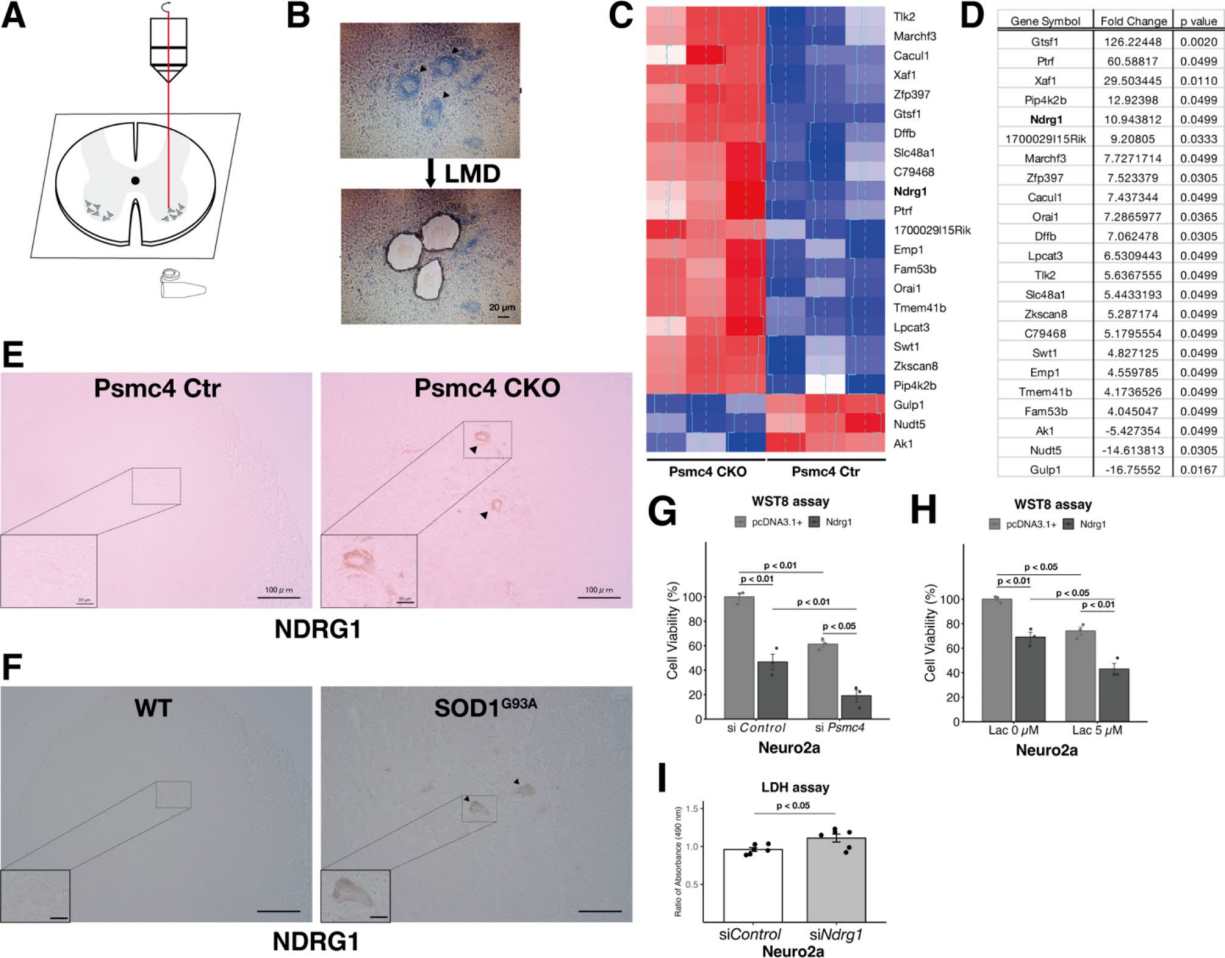
NDRG1 upregulation in Psmc4 CKO mice accelerates cell death in UPS dysfunction. (**A**) Schematic overview of motor neuron capture using AS LMD (Leica). Scale bar: 20 μm. (**B**) The frozen Sect. (10 μm) were stained with 0.1% toluidine blue, and collected motor neurons were analyzed after RNA extraction (TriPure [Roche] and RNeasy micro kit [QIAGEN]) and cDNA synthesis by Ovation Pico WTA System V2 kit (NuGEN Technologies, Inc.). We used samples in which the RNA integrity number was over six and motor neurons were isolated using LMD within 30 min per section. Scale bar: 20 μm. (**C**). Heatmap showing significantly changed genes in motor neurons of Psmc4 CKO (*n* = 3) vs. Ctr (*n* = 3) mice. Data analysis was performed using GeneSpring GX with MAS5 normalization. (**D**) List of identified significantly changed genes. (**E** and **F**) Representative images of immunostaining for NDRG1 in the ventral horns of the lumbar spinal cord of Psmc4 Ctr and Psmc4 CKO mice (**E**) and WT and the end stage of SOD^G93A^ transgenic mice (high copy). (**F**). Arrows indicates motor neurons expressing high levels of NDRG1. Scale bar: 100 μm (low magnification) or 20 μm (high magnification). Fresh frozen (20 μm) were stained using Histofine Simple Stain (NICHIREI BIOSCIENCES INC). The primary antibody used for immunohistochemistry was mouse anti-NDRG1 (1:500; Cell Signaling Technology, RRID: AB_10626626). (**G** and **H**) Neuro2a cells treated with either siControl or siPsmc4 for 72 h (**G**) and lactacystin (Lac; Kyowa Medex) for 24 h (**H**) in the presence of pcDNA3.1 + or pcDNA3.1 + NDRG1 plasmids. Cell viability was measured using the WST8 assay (*n* = 3). one-way analysis of variance (ANOVA) followed by Tukey’s multiple comparison test. (**I**) LDH assay (Dojindo) of Neuro2a cells treated with siControl or siNdrg1 for 72 h. *n* = 6. Student’s t-test. siRNA and plasmid transfection were performed as previously described, with some modifications [15]. Data are presented as the mean ± SEM normalized to the control

To identify novel regulators of UPS dysfunction-induced motor neuron damage, we collected spinal cord motor neurons from 6-week-old male Psmc4 conditional knockout (CKO) (Psmc4 flox/flox; VAChT Cre (+/-)) [6, 7] and control (Ctr) (Psmc4 flox/flox) mice using laser microdissection (LMD) (Fig. 1A and B). Using the approximately 400 motor neurons collected from each genotype, we performed the microarray analysis (Affymetrix GeneChip Mouse Genome 430 2.0 Array), and 23 genes (*Gtsf1*, *Ptrf*, *Xaf1*, *Pip4k2b*, *Ndrg1*, *1700029l15Rik*, *Marchf3*, *Zfp397*, *Cacul1*, *Orai1*, *Dffb*, *Lpcat3*, *Tlk2*, *Slc48a1*, *Zkscan8*, *C79468*, *Swt1*, *Emp1*, *Tmem41b*, *Fam53b*, *Ak1*, *Nudt5*, and *Gulp1*) were significantly changed by more than two-fold in Psmc4 CKO mice (Fig. 1C and D). Immunohistochemical staining showed that N-myc downstream regulated 1 (NDRG1) was upregulated in the motor neurons of Psmc4 CKO mice (Fig. 1E) and the end stage of SOD1^G93A^ mice (22 weeks of age), a mouse model of familial ALS (Fig. 1F).

Next, we examined the pathophysiological function of NDRG1 upregulation in the mouse neuronal cell line Neuro2a (RRID: CVCL_0470). As NDRG1 overexpression forms cleaved caspase 3 products, triggering apoptosis in metastatic cancer cells [8], we examined if high NDRG1 expression could cause cell death. As shown in Fig. 1G and H, NDRG1 overexpression induced cell death in Neuro2a cells. Furthermore, NDRG1 accelerated cell death induced by Psmc4 knockdown (siPsmc4) or well-known proteasome inhibitor lactacystin (Lac) (Fig. 1G and H). Meanwhile, we also conducted NDRG1 knockdown experiments using only siNDRG1 (showing a 71.1% reduction in qRT-PCR; data not shown) and observed cytotoxicity, suggesting that a balance in NDRG1 expression may be important for cell survival (Fig. 1I). NDRG1 expression is induced by p53, a tumor suppressor involved in the caspase-3-dependent apoptotic pathway [8]. The transcription level of p53 increases due to UPS dysfunction in normal human fibroblast cells [9]. This suggests that the p53 pathway mediates NDRG1 upregulation by UPS dysfunction and that NDRG1 accelerates apoptosis. Increased expression of p53 and its activation has been observed in ALS and Alzheimer’s patients [10, 11], while p53 deletion plays a neuroprotective role in Parkinson’s models [12]. Our results suggest that NDRG1 upregulation, potentially due to its acceleration of apoptosis, plays a detrimental role in ALS mouse models. However, another report suggests that the absence of p53 did not alter disease progression in a familial ALS model [13]. Additionally, only a fraction of end-stage SOD1^G93A^ mice exhibited increased NDRG1 expression (data not shown). This may be because cells with upregulated NDRG1 expression were rarely observed in the end stage due to self-induced cell death. Nevertheless, the NDRG1 cell death pathway(s) remain to be elucidated owing to the multifunctional nature of NDRG1. NDRG1 further inhibits autophagy in cancer cells [14]. Normally, when the catabolic process of UPS fails, autophagy is induced to degrade proteins by the autophagy-lysosome pathway. Yet, chronic inhibition of UPS leads to autophagy inhibition, ultimately causing cellular stress, such as aggregate formation—a hallmark of neurodegenerative diseases—and cell death. This indicates that increased NDRG1 expression due to UPS dysfunction may have an important role in shifting cells from living to death conditions as a final cytoprotective mechanism against excessive aggregate formation. Future detailed functional analysis of the cell death pathway induced by NDRG1 is essential.

In conclusion, using transcriptional analysis in motor neurons from Psmc4 CKO mice, we identified NDRG1 as a new modulator of UPS dysfunction, and results reveal that NDRG1 can induce neuronal cell death in vitro. UPS dysfunction is characteristic in patients with neurodegenerative diseases such as Parkinson’s disease, and Alzheimer’s disease, in addition to ALS. Thus, understanding NDRG1’s role in UPS dysfunction might help elucidate the basal mechanism of neurodegenerative diseases like ALS, and contribute to the development of pharmacological treatments.

## Data Availability

The microarray data (CEL files) have been deposited in the public repository under the accession code listed in PRJDB17174. Detailed protocols and reagent information are available from the corresponding author upon request.

## Abbreviations

ALS: Amyotrophic lateral sclerosis
ANOVA: Analysis of variance
CKO: Conditional knockout
Ctr: Control
LMD: Laser microdissection
NDRG1: N-myc downstream regulated 1
SEM: Standard error of the mean
UPS: Ubiquitin-proteasome system
